# Gene expression profiling of human umbilical vein endothelial cells overexpressing CELF2 as diagnostic targets in diabetes-induced erectile dysfunction

**DOI:** 10.3389/fmolb.2025.1596534

**Published:** 2025-07-07

**Authors:** Daniyaer Nuerdebieke, Lizhong Yao, Lange Guo, Jiuzhi Li, Hongliang Jia, Yukui Nan

**Affiliations:** Urology Center, People’s Hospital of Xinjiang Uygur Autonomous Region, Urumqi, China

**Keywords:** diabetes mellitus, erectile dysfunction, RBPs, CELF2, RNA sequencing

## Abstract

**Background:**

Erectile dysfunction (ED) is a common complication of diabetes mellitus (DM), and because of its complex neurovascular etiology, the associated molecular pathogenic mechanisms are not fully understood. This study investigated the important functions and potential molecular regulatory roles of CELF2 in DMED.

**Methods:**

An *in vitro* HUVEC model with CELF2 overexpression was successfully established via transfection with a CELF2-overexpressing lentiviral vector. The effects of CELF2 overexpression on cell proliferation and angiogenesis were assessed via CCK-8 and angiogenesis assays. RNA sequencing was employed to evaluate the gene expression profiles and alternative splicing events regulated by CELF2. An RNA-sequencing assay was performed to evaluate gene expression profiles and alternative splicing genes in HUVECs overexpressing CELF2, and an integration analysis was combined with GSE146078 data to detect potential target genes related to DMED.

**Results:**

The expression of genes related to angiogenesis and the immune response significantly increased with CELF2 overexpression, and the four hub genes associated with alternative splicing in aging and angiogenesis were CXCL2, CXCL10, IL-1A and IL-6.

**Conclusion:**

CELF2 appears to be a key factor in DMED, influencing gene expression and alternative splicing related to angiogenesis and immune responses. The identified hub genes (CXCL2, CXCL10, IL-1A, and IL-6) are closely related to DMED and warrant further investigation to understand the underlying mechanisms and potential therapeutic implications.

## Introduction

It is estimated that by 2030, there will be approximately 643 million diabetic patients worldwide, and the various chronic complications associated with diabetes impose a substantial burden on patients. Diabetes mellitus (DM) is an independent risk factor for erectile dysfunction (ED), with diabetic patients facing a threefold higher probability of developing ED and experiencing its onset approximately 10 years earlier than nondiabetic individuals (International Diabetes Federation, 2021; Cayetano-Alcaraz et al., 2023).

The pathogenesis of diabetes-related ED (DMED) is multifactorial and involves changes in the central nervous system (Lombardi et al., 2015), peripheral nerve activity (Li et al., 2023), and endothelial function (Kwon et al., 2024). Endothelial dysfunction plays a crucial role in the erectile process, and alterations in penile endothelial function are considered to be the cause of both local and systemic vascular lesions (Yao et al., 2022). Moreover, the impaired endothelium leads to increased diabetes and other vascular risk factors, thereby maintaining systemic and penile vascular disease by altering the vascular repair mechanisms of angiogenesis (local repair) and angiogenesis (systemic repair), preventing reendothelialization and angiogenesis (Cannarella et al., 2024; Poredos et al., 2021). Strategies to restore penile endothelial function in diabetes may have significant therapeutic implications for ED associated with both diabetic and nondiabetic vascular disease states. Therefore, there is an urgent need to gain a deeper understanding of its molecular mechanisms to develop more effective treatment methods.

Numerous studies have explored the exact genetic mechanisms of endothelial dysfunction related to ED, but the key genes involved remain unclear. Genome-wide studies have identified dysregulation of inflammatory pathways (e.g., NF-κB signaling) (Jiang et al., 2023), oxidative stress responses (e.g., NRF2 targets) (Ebrahimi et al., 2025) and angiogenic factors (e.g., VEGF & eNOS) (Hu et al., 2021; Yao et al., 2023) in diabetic vascular endothelium. However, upstream RNA-binding proteins orchestrating these transcriptional programs in the context of ED are poorly defined. CUGBP ELAV-like family member 2 (CELF2) is a typical RNA-binding protein that regulates mRNA splicing and stability to ensure intracellular homeostasis (Yanaizu et al., 2020; Itai et al., 2021). It has been reported to regulate RNA splicing and embryonic hematopoietic development and is often dysregulated in neurodevelopmental disorders (Peng et al., 2024), renal tubular epithelial cell apoptosis (Huang et al., 2024), heart failure (Ladd et al., 2005), and diabetes (Gazzara et al., 2017). Previous studies have shown that the expression of the RNA-binding protein CELF2 is downregulated in mouse cavernous endothelial cells (MCECs) stimulated with high glucose, suggesting that CELF2 may play a role in DMED (Yin et al., 2021). However, its specific functions and molecular mechanisms have not been further investigated. In this study, we conducted RNA sequencing experiments to investigate the function and regulatory mechanisms of CELF2 in endothelial cells.

## Materials and methods

### Lentivirus information

CELF2-overexpressing lentivirus was purchased from GenePharma (China, Suzhou). Transcript information: NM_001025077.3, lentiviral vector: LV5 (EF-1a/GFP/Puro/Amp).

### Cell culture and transfections

The HUVEC line (DFSC-EC-01, Zhong Qiao Xin Zhou Biotechnology Co., Ltd., China) was cultured at 37°C with 5% CO_2_ in endothelial cell-specific culture medium (ZQ-1304, Zhong Qiao Xin Zhou Biotechnology Co., Ltd., China) supplemented with 10% fetal bovine serum (FBS) (10099–141, Gibco, China), 100 μg/mL streptomycin, and 100 U/mL penicillin (SV30010, HyClone, United States).CELF2-overexpressing HUVECs were generated via lentiviral transduction (MOI = 35), followed by puromycin selection (1 μg/mL) for stable clone establishment, and the cells were harvested for RT‒qPCR and Western blot analysis.

### Assessment of gene expression

cDNA synthesis was performed with a reverse transcription kit (R323-01, Vazyme, China) at 42°C for 5 min, 37°C for 15 min, and 85°C for 5 s on a thermocycler (T100, Bio-Rad, United States). qPCR was performed on an ABI QuantStudio 5, followed by denaturation at 95°C for 10 min, 40 cycles of denaturation at 95°C for 15 s and annealing and extension at 60°C for 1 min. Each sample had three technical replicates. The concentration of each transcript was then normalized to that of glyceraldehyde-3-phosphate dehydrogenase (GAPDH), and the mRNA level was determined via the 2^−ΔΔCT^ method for analysis (Livak and Schmittgen 2001). Comparisons were performed with paired Student’s t tests via GraphPad Prism software (version 8.0, San Diego, CA). The primers used for quantitative (q)PCR analysis are presented in Table 1.

**TABLE 1:** Primer sets related to the experimental procedures.

Gene	Primer	Sequence (5′-3′)
GAPDH	Forward	GGTCGGAGTCAACGGATTTG
	Reverse	GGAAGATGGTGATGGGATTTC
CELF2	Forward	AGAAGGAAGGTCCAGAGG
	Reverse	GCACTTGCTCAGATTGGT

### Western blot

HUVECs were lysed in ice-cold RIPA buffer (PR20001, Proteintech, China) supplemented with a protease inhibitor cocktail (4693116001, Sigma, United States) and incubated on ice for 30 min. The samples were boiled for 10 min in boiling water with protein loading buffer (P1040, Solarbio, China), loaded onto a 10% SDS‒PAGE gel and transferred onto 0.45 mm PVDF membranes (ISEQ00010, Millipore, United States). The PVDF membranes were then blocked for 1 h at room temperature and incubated overnight at 4°C with primary antibodies against Flag (anti-Flag, 1:1,000, antibody produced in mouse, 66008-3-Ig, Proteintech, China) and GAPDH (1:5,000, antibody produced in mouse, 60004-1-Ig, Proteintech, China), followed by incubation with horseradish peroxidase-conjugated secondary antibodies (anti-mouse, 1:10,000, AS003, ABclonal, China) for 45 min at room temperature. Then, the membranes were visualized via chemiluminescence with enhanced ECL reagent (P0018FM, Beyotime, China).

### CCK-8

CELF2-overexpressing HUVECs and control cells were seeded into a 96-well culture plate at 100 μL per well at a density of 3000–5000 cells per well. At least 6 replicate wells were used for each group for statistical analysis. The 96-well plate was placed in a cell culture incubator at 37°C and 5% CO_2_ for incubation until the cells adhered and grew to 70%–80% confluence. On the day of the experiment, 10 μL of CCK-8 reagent was added to each well, and the plate was gently shaken to ensure even distribution of the reagent. The plate was then returned to the cell culture incubator for incubation for 1–4 h to allow the CCK-8 to react with the cellular metabolic products. The absorbance (OD value) of each well was measured at a wavelength of 450 nm via an enzyme-labeled instrument. The enzyme-labeled instrument was calibrated and ready. The cell proliferation rate was calculated on the basis of the absorbance values.

### Tube formation assay

A 96-well plate was used, and 50 μL of matrix gel (356234, Corning, United States) was carefully added to each well to ensure complete coverage of the well bottom without producing bubbles. The 96-well plate was then placed in an incubator at 37°C for 30 min to allow the matrix gel to solidify. The cells were rinsed with PBS and digested with an appropriate amount of trypsin. The digestion was stopped by adding termination solution. After centrifugation at 1,000 rpm for 5 min, the supernatant was discarded, and the cells were resuspended in 1 mL of complete medium, counted, and adjusted to a final concentration of 10,000 cells/50 μL. After the matrix gel solidified, the plate was removed from the incubator, and 50 μL of cell suspension was added to each well. In general, the cells began to form tubular structures within 2–4 h.

### RNA-seq and analysis

Strand-specific cDNA libraries were prepared from total RNA of CELF2-overexpressing and control HUVECs using the NEBNext Ultra II RNA Library Prep Kit and sequenced on an Illumina NovaSeq 6000 platform (paired-end, 150 bp). Raw reads underwent quality control via FastQC, followed by adapter/low-quality base trimming with Trimmomatic (parameters: LEADING:20, TRAILING:20, SLIDINGWINDOW:4:20, MINLEN:50). Clean reads were aligned to the human reference genome (GRCh38) using HISAT2 and quantified via featureCounts (Ensembl v105 annotation).

Gene expression levels were normalized as FPKM to account for transcript length and sequencing depth. Differential expression analysis was performed using DESeq2 (adjusted p < 0.05, |log2 (fold change)| > 1). Principal component analysis (PCA) and hierarchical clustering were conducted on variance-stabilized counts. Sample correlations were assessed using Pearson’s coefficient, and heatmaps visualized expression patterns of differentially expressed genes (DEGs).

For transcriptome assembly, uniquely mapped reads were analyzed with StringTie, and novel transcripts were identified via Cuffcompare. Coverage uniformity across transcripts and genes was evaluated by dividing sequences into 100 bins and calculating read density. TSS/TTS-proximal read distributions (±1 kb) were also quantified.

Gene Ontology (GO) and KEGG pathway enrichment analyses were performed using clusterProfiler, with hypergeometric testing (q < 0.05) to identify top 10 enriched terms. Alternative splicing events were detected via rMATS (FDR <0.05, |ΔPSI| > 0.1), and Circos visualized genome-wide read depth distribution.

### Alternative splicing analysis

The alternative splicing events (ASEs) and regulated alternative splicing events (RASEs) between the samples were defined and quantified by using the ABL pipeline as described previously (Jin et al., 2017; Xia et al., 2017). In brief, ABL for the detection of ten types of ASEs was based on splice junction reads, including exon skipping (ES), alternative 5′splice site (A5SS), alternative 3’s splice site (A3SS), mutually exclusive exons (MXE), mutually exclusive 5′UTRs (5pMXE), mutually exclusive 3′UTRs (3pMXE), cassette exons, A3SS&ES and A5SS&ES.

To assess RBP-regulated ASE, Student’s t-test was performed to evaluate the significance of the ratio alteration of AS events. Those events that were significant at the P value cutoff corresponding to a false discovery rate cutoff of 5% were considered RBP-regulated ASEs.

The Search Tool for the Retrieval of Interacting Genes (STRING) database (http://stringdb.org/) (Szklarczyk et al., 2019) was used to construct protein‒protein interaction (PPI) networks by mapping the DEGs to PPI data. The symbols of the DEGs were imported into the database, and high-resolution bitmaps were generated. The bitmap included only interactors with a combined confidence score of 0.4 or higher. The hotspot module was obtained from large PPI networks via Molecular Complex Detection (MCODE), which is a Cytoscape plugin. In this study, the MCODE parameters were as follows: degree of cutoff = 2; cluster finding, haircut; node score cutoff = 0.2; k-core = 2; and maximum depth = 100. CytoHubba, a plugin for Cytoscape 3.9.1, was used to calculate the degree of connectivity and identify the top 8 genes in the PPI networks (Liu et al., 2022). The PPI networks, modules, and top 8 genes were visualized on the basis of their node degree via Cytoscape software.

### Statistical analysis

All the data are expressed as the means ± standard errors. Statistical analysis was performed via the Mann–Whitney U test. All p values less than 0.05 were considered significant.

## Results

### Establishment of a CELF2-overexpressing HUVEC model via lentiviral transfection

**FIGURE 1:**
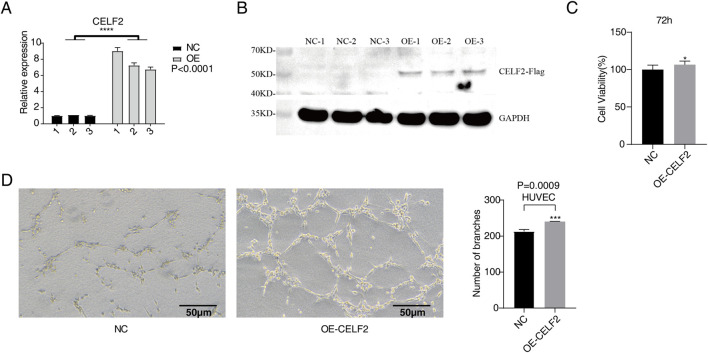
CELF2 overexpression accelerates the proliferation and angiogenesis of HUVECs. **(A)** The histogram shows the RT‒qPCR results of CELF2-overexpressing HUVECs. The error bars represent the means ± SEMs. ****P value <0.0001. **(B)** Western blot analysis revealed that CELF2 was successfully overexpressed in HUVECs. **(C)** The histogram shows the proliferation of CELF2-overexpressing cells after 72 h. **(D)** The diagram shows the angiogenic experimental results of CELF2 overexpression after 20 h. The left panel shows the results of angiogenesis in cells, and the right panel shows the quantitative results of CELF2 overexpression after 20 h.

In this study, we established CELF2-overexpressing HUVEC lines via the use of recombinant lentiviral vectors. CELF2 expression levels were assessed 24 h posttransfection through RT‒qPCR and Western blot analyses. The results revealed that CELF2 mRNA expression in lentivirus-infected HUVECs overexpressing CELF2 was 6- to 8-fold higher than that in their control vector-infected counterparts (P < 0.001), demonstrating the efficacy of lentivirus-mediated CELF2 overexpression. Protein-level analysis further confirmed a significant increase in CELF2 expression in the overexpression group compared with the control group (P < 0.05), confirming the successful construction of a stable HUVEC model with lentivirus-driven CELF2 overexpression (Figures 1A,B).

### Evaluation of the effects of CELF2 overexpression on cell proliferation and angiogenesis

To evaluate the effect of CELF2 overexpression on the proliferative capacity of human umbilical vein endothelial cells (HUVECs), we performed a CCK-8 cell proliferation assay 72 h after lentiviral infection. The results demonstrated that the CELF2-overexpressing group exhibited significantly greater cell viability than the control group did (P < 0.05), indicating that CELF2 effectively promoted HUVEC proliferation (Figure 1C). In the Matrigel-based *in vitro* angiogenesis assay, the number of tubular structures formed by CELF2-overexpressing HUVECs was markedly greater than that formed by control HUVECs (P < 0.005), suggesting that CELF2 enhances the angiogenic potential of HUVECs (Figure 1D).

### Overview of the RNA-sequencing data

**FIGURE 2:**
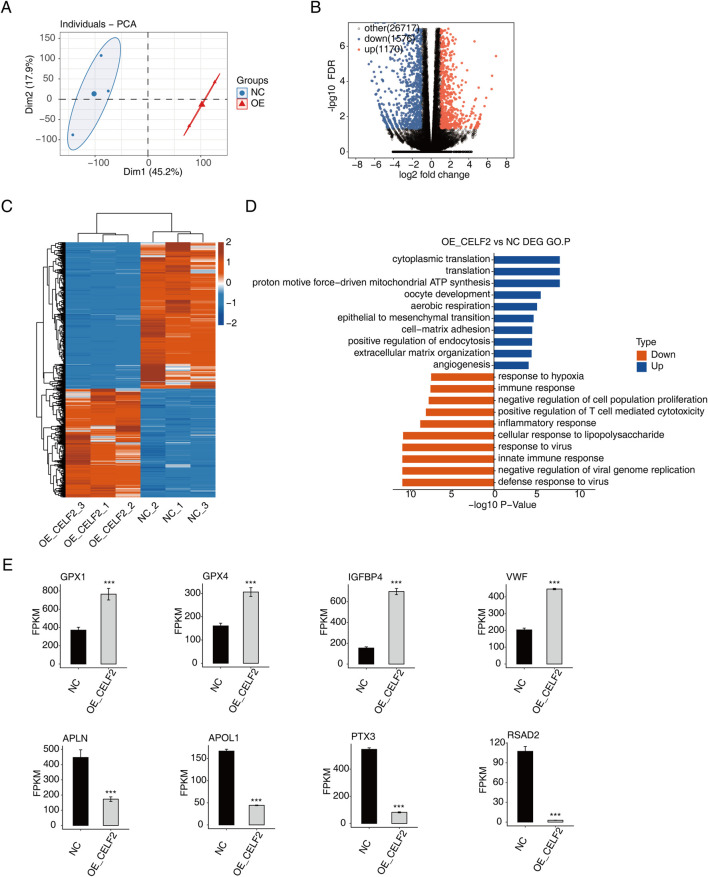
CELF2 regulates gene expression in HUVECs. **(A)** PCA based on the FPKM values of all detected genes. The ellipse for each group is the confidence ellipse. **(B)** Volcano plot showing all differentially expressed genes (DEGs) between the OE-CELF2 and NC samples. **(C)** Hierarchical clustering heatmap showing the expression levels of all DEGs. **(D)** Histogram showing the most enriched GO biological process results for the upregulated genes and downregulated genes. **(E)** Bar plot showing the expression patterns and significant differences in DEGs among the four upregulated genes and four downregulated genes. The error bars represent the means ± SEMs. ***P value <0.001. **P value <0.01. *P value <0.05.

In this study, we constructed sequencing libraries for RNA-seq analysis from CELF2-overexpressing and control groups (n = 3 per group). Principal component analysis (PCA) revealed significant separation between the two groups (Figure 2A). A total of 2,746 genes were detected across both libraries, with 1,170 upregulated and 1,576 downregulated genes identified (Figures 2B,C). Figure 2D lists the top 10 enriched activated pathways and 10 suppressed pathways, including biological processes such as cytoplasmic translation, translation, proton-driven mitochondrial ATP synthesis, oocyte development, aerobic respiration, epithelial‒mesenchymal transition, cell‒matrix adhesion, positive regulation of endocytosis, extracellular matrix organization, and angiogenesis. Among the upregulated and downregulated genes in CELF2-overexpressing HUVECs, the expression levels of CXCL2, CXCL10, IL1A, IL-6, ECSCR, CIB1, EFNA1, and EMC10 were significantly different (Figure 2E). These genes may regulate proangiogenic and erectile functions at the transcriptional level.

### CELF2 influences alternative splicing of genes in HUVECs

**FIGURE 3:**
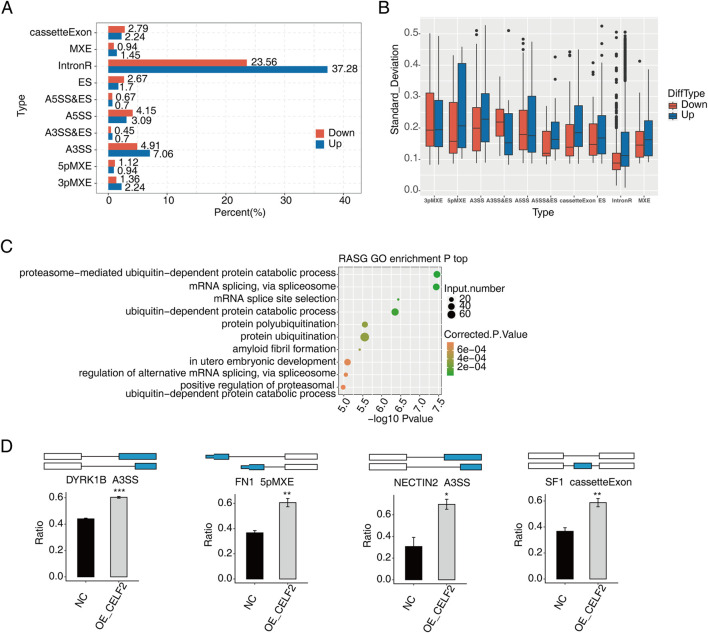
CELF2 influences alternative splicing of genes in HUVECs. **(A)** Bar plot showing the percentages of each type of upregulated or downregulated CELF2-regulated alternative splicing event (RASE). **(B)** Boxplot showing the standard deviation of the ratio of each type of CELF2-regulated alternative splicing event (RASE) in all samples. **(C)** Scatter plot showing the most enriched GO biological process results of the regulated alternative splicing genes (RASGs). **(D)** Schematic diagrams depicting the regulation of alternative splicing genes by CELF2. The error bars represent the means ± SEMs. ***P value <0.001. **P value <0.01. *P value <0.05.

To systematically characterize abnormal AS events (ASEs) following CELF2 overexpression, we compared PSI values between HUVECs with CELF2 overexpression and matched normal cells. Among the ten differential splicing events that we evaluated, ASEs were most frequent for introns and cassette exons, followed by AESSs and A3SSs (Figures 3A,B), and most ASEs involved ES, an A5SS or an A3SS. Additionally, we observed that the numbers of AS events with increased vs decreased PSI values were similar, with the exception of RI and AD events. We further conducted GO analysis on genes associated with CELF2-regulated AS events in HUVECs. The genes that underwent alternative splicing were most commonly enriched in embryo development *in utero*, proteasome-mediated catabolic processes, transcriptional regulation and related pathways (Figure 3C). Genes whose expression is affected by transcription factors (TFs) in which alternative splicing events have been identified, including DYRK1B, FN1, NECRIN2, and SF1, may be possible targets for the treatment of diabetic vascular disease (Figure 3D).

### Verification of key target gene expression of CELF2

**FIGURE 4:**
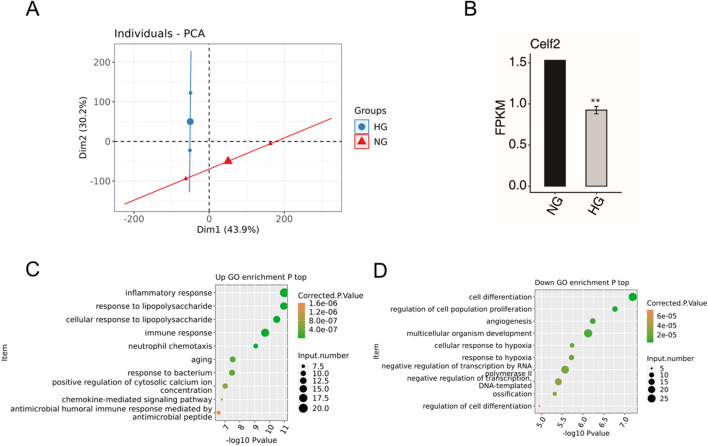
DEG Data of GSE146078. **(A)** PCA based on the FPKM values of all detected genes. The ellipse for each group is the confidence ellipse. **(B)** Bar plot showing the expression pattern and significant difference in Celf2 expression. The error bars represent the means ± SEMs. ** P value < 0.01. **(C)** Scatter plot showing the most enriched GO biological process results for the upregulated genes. **(D)** Scatter plot showing the most enriched GO biological process results for the downregulated genes. The error bars represent the means ± SEMs. ** P value < 0.01.

To further identify DMED-related differential genes and variable splicing events potentially regulated by CELF2. The RNA-seq data of GSE146078 were downloaded and included 2 samples of mouse cavernous endothelial cells (MCECs) exposed to normal glucose (NG) and high glucose (HG) conditions. In total, 23,282 genes were detected in both libraries, including 170 upregulated genes and 1576 downregulated genes (Figure 4A). The upregulated DEGs were enriched mainly in the inflammatory response, immune response, aging and related pathways (Figure 4C). For the downregulated DEGs, the enriched GO terms were cell differentiation, angiogenesis, hypoxia and related biological processes (Figure 4D). Moreover, the HG group presented significantly reduced CELF2 expression, reflecting high glucose-induced downregulation of CELF2 and suggesting its critical role in diabetes-associated erectile dysfunction (ED) (Figure 4B).

**FIGURE 5:**
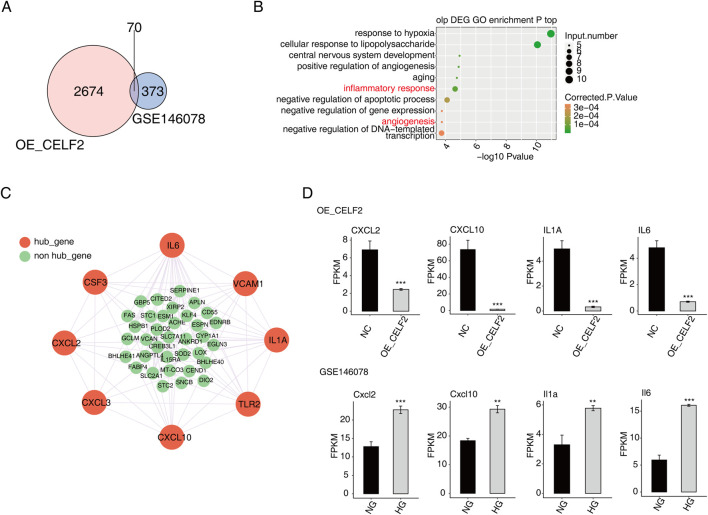
Verification of key target gene expression of CELF2. **(A)** Venn diagram showing the overlapping DEGs between HUVECs and GSE146078. **(B)** Scatter plot showing the most enriched GO biological process results of the overlapping DEGs. **(C)** The network diagram shows the hub genes selected from the overlapping DEGs through the PPI (confidence level 0.4) and MCODE interaction analyses. **(D)** Bar plot showing the expression patterns and significant differences of the overlapping DEGs. The error bars represent the means ± SEMs. ***P value <0.001. **P value <0.01. *P value <0.05.

Moreover, the potential DMED-related genes and alternative splicing events potentially regulated by CELF2 were analyzed via integration with CELF2-overexpressing HUVEC RNA-seq data. A total of 70 overlapping genes were found, of which 25 were upregulated and 45 were downregulated (Figure 5A). The genes most commonly enriched in hypoxia, the cellular response to lipopolysaccharide, central nervous system development, angiogenesis, aging, the inflammatory response and other related biological pathways (Figure 5B). Among the many upregulated genes, the ECSCR, CIB1, EFNA1, EMC10, RAMP2, and DLL4 genes were closely related to angiogenesis (Supplementary Figure S1). Furthermore, on the basis of the STRING database and Cytoscape software, a PPI network consisting of 43 nodes and 101 edges was constructed (Figure 5C).

The majority of the genes that met the filter settings in the STRING database were upregulated. Four modules were selected after MCODE analysis (Supplementary Figure S2). With the highest score of 8, module 1 was significantly enriched in the inflammatory response and angiogenesis. As important components of module 1, eight genes (CXCL2, CXCL3, CXCL10, TLR2, IL-1A, VCAM1, IL-6 and CSF3) were identified as hub genes of the whole network, and four of the hub genes (CXCL2, CXCL10, IL-1A and IL-6) were confirmed to have significant differential expression and were closely related to DMED (Figure 5D).

## Discussion

Endothelial dysfunction is recognized as a mainstay in the pathophysiology of diabetic ED disease and is induced by the detrimental actions of high glucose levels and increased oxidative stress on endothelial cells that make up the vascular lining (Defeudis et al., 2022). Currently, the corpus cavernosum at the tissue and cellular levels is the most studied, but the molecular pathways involved in endothelial dysfunction are not fully understood, which may improve the understanding of the triad of endothelial dysfunction in patients with DMED by assisting in the identification of novel therapeutic targets. Alternative splicing is a conserved biological process that diversifies the transcriptome and proteome and plays a key role in gene expression, in which introns within nascent RNA are removed and exons are ligated to form mature mRNAs (Lai et al., 2022). Dlamini et al. proposed that studies have focused on splicing variants of genes involved in apoptosis pathways and genes involved in insulin resistance and obesity and demonstrated the importance of variable splicing subtypes of these genes that may play a role in diabetes (Dlamini et al., 2019). Previously, the expression level of CELF2 in MCECs was significantly downregulated after high glucose treatment, suggesting that CELF2 may play a potential role in DMED. Here, we overexpressed CELF2 in HUVECs, performed an RNA-sequencing assay to detect its function in cell proliferation, apoptosis and angiogenesis, and investigated potential target genes for ED.

The significant increase in CELF2 mRNA and protein levels following lentivirus infection indicates that CELF2 is a suitable model for studying its effects on HUVECs, particularly in promoting cell proliferation and angiogenesis. GO analysis of the RNA-sequencing results revealed that the genes correlated with CELF2, which was significantly altered, were enriched in angiogenesis and the immune response. Among the many upregulated genes, the ECSCR, CIB1, EFNA1, EMC10, RAMP2, and DLL4 genes were found to be closely related to angiogenesis. In addition, significantly downregulated genes were enriched in viral genome replication, the innate immune response and the inflammatory response, suggesting that the immune system may play a potent pathogenic role in the development of DMED.

The CELF2-related AS events of NECTIN2, DYRK1B, SF1 and FN1 were significantly different and may be potential therapeutic targets for diabetes-related vascular disease. To further investigate the DMED-related target genes regulated by CELF2. We performed an integrated analysis of our RNA-seq data and the GEO open database concerning the DMED of GSE146078, and the overlapping genes were enriched in hypoxia, angiogenesis and aging.

Our integrated analysis identified CXCL2, CXCL10, IL-1A, and IL-6 as hub genes whose dysregulation may mediate CELF2’s effects in DMED. Through integrated analysis, this study identified four hub genes—CXCL2, CXCL10, IL-1A, and IL-6—whose aberrant expression may mediate the role of CELF2 in DMED. As chemokines, CXCL2 and CXCL10 are overexpressed in the diabetic vascular microenvironment. They exacerbate chronic inflammation and oxidative stress in the corpus cavernosum by recruiting neutrophils/monocytes, thereby impairing endothelium-dependent vasodilation (Szpakowska et al., 2023). Previous studies indicate that IL-6 inhibits the Wnt/β-catenin pathway via STAT3 activation (Yoshida, 2020), while Wnt signaling is critical for maintaining vascular homeostasis in the corpus cavernosum: Wnt5a promotes endothelial cell proliferation and angiogenesis, whereas loss of β-catenin induces apoptosis of cavernous smooth muscle cells (Gu et al., 2021). Notably, IL-6 downregulates eNOS phosphorylation in cavernous tissue, reducing NO production and contributing to ED pathogenesis (Wang et al., 2020). These findings may have clinical implications for the early diagnosis of vascular disease caused by aging and angiopathy.

Beyond dysregulation of the identified hub genes (CXCL2, CXCL10, IL-1A, IL-6), lipid raft microdomains—cholesterol- and sphingolipid-enriched membrane platforms—may spatially orchestrate their receptor signaling in diabetic endothelium. As highlighted in recent advances (Warda et al., 2025), lipid rafts cluster inflammatory receptors (e.g., IL-6R, CXCR3) and downstream effectors to amplify signaling cascades. In diabetes, hyperglycemia disrupts raft integrity by altering cholesterol distribution, impairing NO synthesis and promoting endothelial dysfunction. Given that CELF2 regulates key inflammatory mediators implicated in DMED, its potential influence on raft-associated receptor localization warrants investigation. Therapeutic strategies targeting raft dynamics (e.g., cholesterol modulators) show promise in restoring vascular homeostasis in metabolic disorders.

To our knowledge, this is the first study to perform systematic profiling of gene alterations and alternative splicing events related to CELF2 in HUVECs. However, our study has several limitations. First, CELF2 overexpression in HUVECs did not completely mimic the expression level of DMED. Second, while RNA sequencing identified key differentially expressed genes and alternative splicing events regulated by CELF2, we did not perform Western blot analysis to confirm corresponding protein expression changes. This includes critical endothelial markers such as VEGF and eNOS, which are functionally relevant to angiogenesis and erectile function. The absence of protein-level data limits mechanistic interpretations of how transcriptional changes translate to functional outcomes. Third, the CELF2-overexpressing HUVEC model provides insights into molecular functions but does not fully replicate the complex pathophysiology of DMED. And several significantly altered genes identified by RNA-seq were not validated by RT-qPCR due to resource constraints. We will prioritize protein-level validation of hub genes (CXCL2, CXCL10, IL-1A, IL-6) and endothelial markers (VEGF, eNOS) in future work.

## Conclusion

We profiled differentially expressed genes (DEGs) in MCEC HUVECs overexpressing CELF2. In the analysis of GO categories, we found that angiogenesis and the immune response had the highest proportions of genes. CELF2 influences the alternative splicing of genes related to aging and angiogenesis, and four hub genes (CXCL2, CXCL10, IL-1A and IL-6) were confirmed to be significantly differentially expressed and closely related to DMED; additional research is necessary to elucidate the potential mechanisms involved in diabetes-induced ED.

## Data Availability

The data presented in the study are deposited in the GEO repository, accession number GSE293115.
