# Supplementary material for: Gene expression profiling of human umbilical vein endothelial cells overexpressing CELF2 as diagnostic targets in diabetes-induced erectile dysfunction
Source: Front Mol Biosci. 2025 Jul 7;12:1596534. doi: 10.3389/fmolb.2025.1596534 (PMC12277130; doi:10.3389/fmolb.2025.1596534)
Supplement: Supplementary file 1 [file Supplementaryfile1.docx]

**Gene expression profiling of human umbilical vein endothelial cells with CELF2 overexpression for diagnostic targets in diabetes-induced erectile dysfunction**

**Supplementary figures：Figure S1-S2**


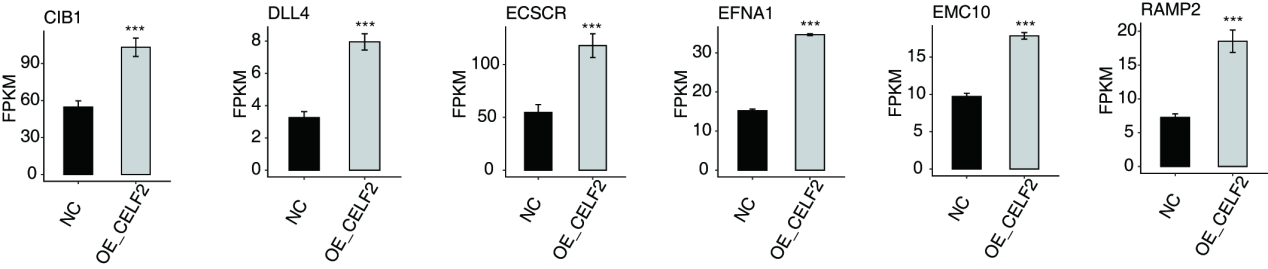


FigS1 (A)The Bar plot showing the expression pattern and statistical difference of DEGs associated with angiogenesis. Error bars represent mean ± SEM.*** P-value < 0.001.** P-value < 0.01.* P-value < 0.05.


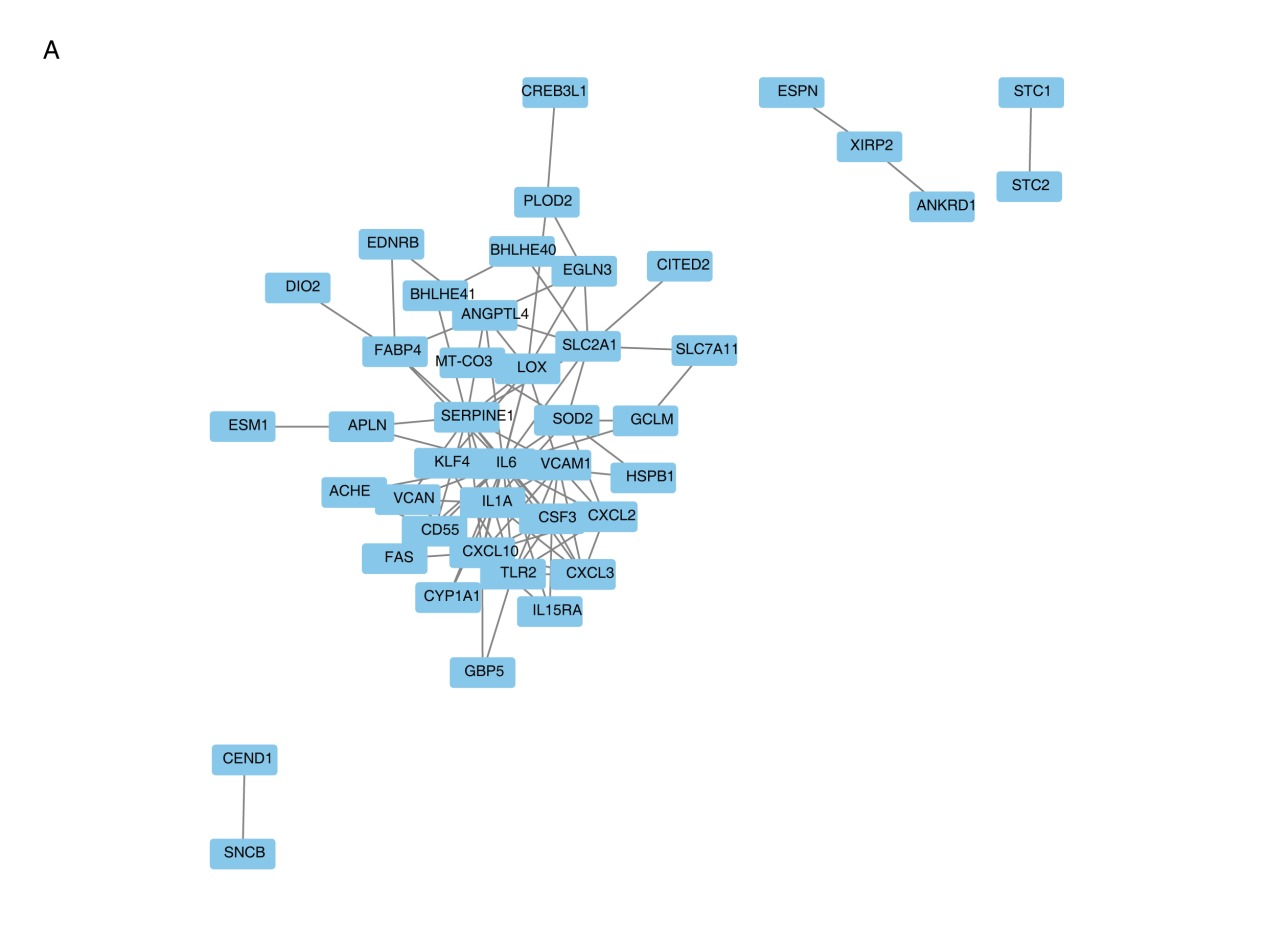


**Fig S2. PPI (confidence level 0.4) and MCODE interaction analysis.**
